# The relationship between metabolic syndrome and asthma in the elderly

**DOI:** 10.1038/s41598-018-26621-z

**Published:** 2018-06-20

**Authors:** Sangshin Park, Nam-Kyong Choi, Seungsoo Kim, Chang-Hoon Lee

**Affiliations:** 1 Center for International Health Research, Rhode Island Hospital, The Warren Alpert Medical School of Brown University, Providence, Rhode Island United States; 2 Department of Pediatrics, The Warren Alpert Medical School of Brown University, Providence, Rhode Island United States; 3 Department of Health Convergence, Ewha Womans University, Seoul, Republic of Korea; 4 Division of Allergy and Pulmonology, Department of Internal Medicine, Catholic University of Korea Daejeon St. Mary’s Hospital, Daejeon, Republic of Korea; 5 Division of Pulmonary and Critical Care Medicine, Department of Internal Medicine, Seoul National University Hospital, Seoul, Republic of Korea

## Abstract

The burden of asthma in the elderly is increasing, but the etiology of asthma in the elderly is not clearly understood. Recent studies have reported the epidemiological link between metabolic syndrome (MS) and asthma, but it has rarely been studied in the elderly. This study investigated the association between MS and asthma and the contribution of insulin resistance (IR) and systemic inflammation to this MS-asthma association in the elderly. Our study analyzed 4,060 elderly participants (≥65 years old) from a cross-sectional survey, the Korean National Health and Nutritional Examination Survey 2007–2012. Mediation analyses were performed to examine whether IR and systemic inflammation mediates the MS-asthma association. Participants with MS had significantly higher prevalence of asthma (adjusted odds ratio = 1.34; 95% confidence interval = 1.09–1.64), and those who had greater waist circumference and lower HDL-C were especially likely to have asthma. Participants with IR and systemic inflammation were associated with higher prevalence of asthma. Prevalence of IR and systemic inflammation were higher in participants with MS or with each MS component. The MS-asthma association was substantially mediated by IR and systemic inflammation. Our study showed a significant association between MS and asthma in the elderly. MS might affect asthma through both IR and systemic inflammation.

## Introduction

During the past decades, asthma has become one of the major health burdens on both individuals and society. It is estimated that approximately 300 million people worldwide currently have asthma and that the number will increase to 400 million by 2025^1^. Although asthma was regarded as a disease for children and young adults in the past, it has been recently highlighted that asthma is also common in elderly people of age 65 or older. The prevalence of asthma in the elderly has been reported as 4.5%^2^ to 12.7%^3,4^, and approximately 50% of deaths from asthma occur in this age group^5,6^. With the rapid aging of the global population, the burden of asthma on the elderly will further increase.

The pathophysiology and risk factors of asthma in the elderly are not clearly understood. It has been questioned whether asthma in the elderly should be considered as the same disease as asthma in children and younger adults^7^. While investigators observed clinical and physiological similarities between asthma in the elderly and that in younger patients^7^, several studies also reported that the characteristics of asthma in the elderly were distinct from the disease when found in young adults^8^. Some studies reported that patients with late-onset asthma show lower prevalence of positive skin test response when compared with early-onset asthmatics^9^. Such results suggest that non-allergic mechanisms might contribute more substantially to the development of asthma in the elderly.

Metabolic syndrome (MS) is another crucial medical condition in the elderly. MS is a risk factor for cardiovascular morbidity and mortality, and the prevalence of MS in the elderly ranges from 11% to 55% in different studies^10,11^. Recently, the epidemiological link between MS and asthma has been well studied. For example, a prospective cohort study (HUNT) that included 23,191 adults of age 19 to 55 years showed that a person with MS had a 57% higher risk of asthma incidence during an average of 11 years of follow-up^12^. However, the association between MS and asthma in the elderly has rarely been investigated. For example, the HUNT study excluded those of age 65 years and above at follow-up to reduce the misclassification bias of asthma and chronic obstructive pulmonary disease^12^.

The aims of our study were to (1) investigate the associations between MS and asthma and (2) examine whether potential mediators, such as insulin resistance (IR) and systemic inflammation, mediate this MS-asthma association in Korean elderly participants of a nationwide survey.

## Results

Participants with MS tended to be retired rural residents with lower level education and higher household income compared to those without MS (Table 1). They were less likely to exercise, more likely to smoke, and had worse metabolic profiles.

**Table 1:** Characteristics of participants according to MS.

	MS: no	MS: yes^*^	*P* value
N (%)	2072 (51.0)	1988 (49.0)	
Women, %	75.0	86.4	<0.0001
Age, %			
65–69	38.5	39.5	0.378
70–74	31.2	32.2	
≥75	30.3	28.3	
Social economic status, %			
House hold income ≥$2,000/month	25.0	27.9	0.042
Education ≥middle school	24.5	21.7	0.039
Still working	40.2	28.9	<0.001
Urban resident	45.0	35.9	<0.001
Lifestyle, %			
Physical activity ≥1,000METs/week	53.8	46.7	<0.001
Ever-smoker	15.1	9.6	<0.001
Involuntary smoker	17.3	17.8	0.693
Anthropometric and biological variable			
Continuous variable			
WC, cm	79.4 ± 0.2	87.8 ± 0.2	<0.001
SBP, mmHg	127.5 ± 0.4	134.1 ± 0.4	<0.001
DBP, mmHg	75.1 ± 0.2	77.2 ± 0.2	<0.001
TG, mg/dL	106.7 ± 1.1	174.8 ± 2.4	<0.001
HDL- C, mg/dL	53.2 ± 0.3	44.9 ± 0.2	<0.001
FG, mg/dL	96.1 ± 0.4	110.1 ± 0.6	<0.001
Insulin, μU/mL	8.6 ± 0.1	11.7 ± 0.2	<0.001
HOMA-IR	2.06 ± 0.04	3.24 ± 0.06	<0.001
WBC count, 1,000/μL	5.71 ± 0.04	6.29 ± 0.04	<0.001
Binary variable, %			
Asthma	10.3	13.5	0.002
IR (HOMA-IR: > 3.06)	10.6	39.9	<0.001
Systemic inflammation (WBC count: > 6.83 × 1,000/μL)	18.9	31.2	<0.001

Data are presented as % or mean ± SE. *P* values derived from Student t test and Chi-square test. *MS was defined according to the Joint Interim Statement clinical criteria with Korean-specific abdominal obesity criteria. MS, metabolic syndrome; WC, waist circumference; SBP, systolic blood pressure; DBP, diastolic blood pressure; TG, triglyceride; HDL-C, high-density lipoprotein cholesterol; FG, fasting glucose; HOMA-IR, homeostasis model assessment of insulin resistance (IR); WBC, white blood cell.

**Figure 1:**
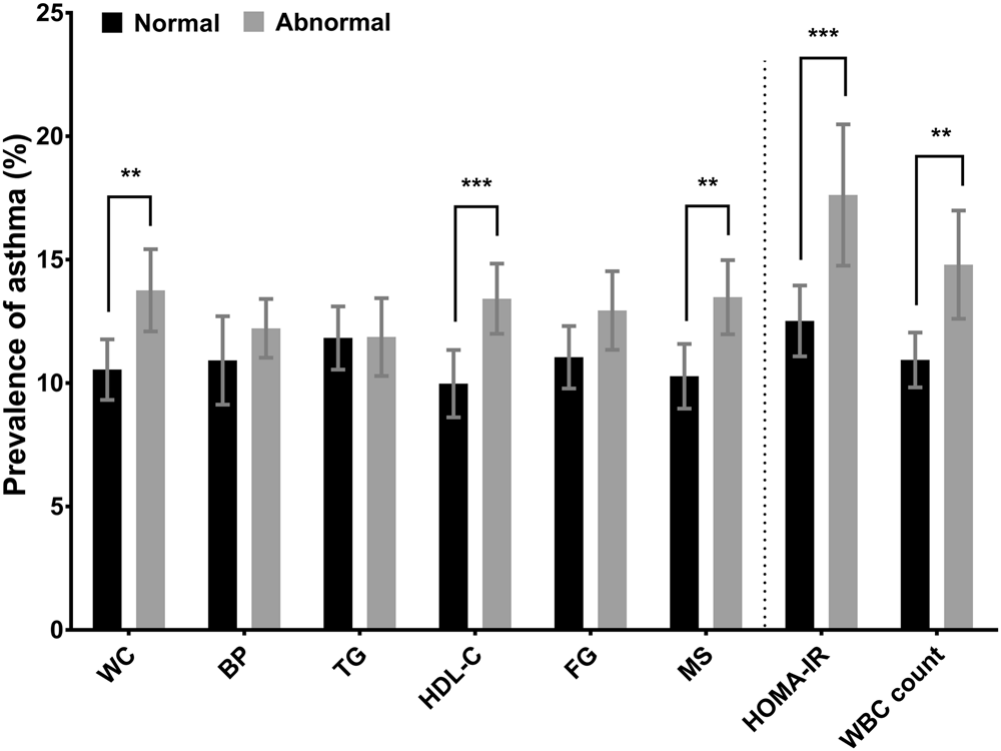
Prevalence of asthma according to the status of metabolic syndrome (MS), MS components, homeostasis model assessment of insulin resistance (HOMA-IR), and white blood cell (WBC) count. MS and MS components were defined according to the Joint Interim Statement clinical criteria with Korean-specific abdominal obesity criteria. Abnormal HOMA-IR, termed “insulin resistance,” was determined when HOMA-IR was greater than 3.06 (75th percentile). Abnormal WBC count, termed “systemic inflammation,” was determined when the WBC count greater than 6,830/µL (>75th percentile). The error bars represent 95% confidence intervals. *P* values were derived from Chi-square test. WC, waist circumference; BP, blood pressure; TG, triglyceride; HDL-C, high-density lipoprotein cholesterol; FG, fasting glucose. **P value < 0.01; ***P value < 0.001.

Participants with MS had significantly higher prevalence of asthma compared to those without MS (Fig. 1). The average onset age (±SE) of asthma in elderly was 60.3 ± 1.59 years (58.7 ± 2.18 years in asthmatics with MS; 64.7 ± 1.76 years in asthmatics without MS). MS was significantly associated with asthma even after adjustment by covariates in multivariable regression models (adjusted odds ratio = 1.34; 95% confidence interval = 1.09–1.64) (Table 2). Among MS components, abdominal obesity (or having greater waist circumference) and low high-density lipoprotein cholesterol (HDL-C) were significantly associated with asthma. IR and systemic inflammation were also significantly associated with asthma (Fig. 1; Table 2). The prevalence of asthma rose with an increasing number of MS components (*P* for trend < 0.01, data not shown). MS and each MS component were significantly associated with IR and systemic inflammation (Table 2).

**Table 2:** Associations among MS, its components, HOMA-IR, IR, WBC count, systemic inflammation, and asthma.

Predictor	Outcome				
	HOMA-IR (per 1 unit)	IR^‡^	WBC count (per 1,000/μL)	Systemic inflammation^§^	Asthma
MS^†^	1.13 (0.99, 1.27)	5.37 (4.33, 6.65)	0.58 (0.47, 0.68)	1.97 (1.68, 2.30)	1.34 (1.09, 1.64)
**MS component: continuous variable**					
WC (per 10 cm)	0.64 (0.57, 0.72)	2.52 (2.24, 2.83)	0.28 (0.22, 0.33)	1.29 (1.19, 1.39)	1.27 (1.14, 1.41)
SBP (per 10 mmHg)	NS	1.08 (1.02, 1.13)	0.05 (0.02, 0.08)	1.05 (1.01, 1.09)	NS
DBP (per 10 mmHg)	NS	NS	0.06 (0.00, 0.11)	NS	NS
TG (per 10 mg/dL)	0.03 (0.02, 0.04)	1.04 (1.03, 1.06)	0.03 (0.02, 0.04)	1.04 (1.03, 1.05)	NS
HDL-C (per 10 mg/dL)	−0.10 (−0.17, −0.04)	0.82 (0.75, 0.89)	−0.09 (−0.14, −0.05)	0.88 (0.82, 0.94)	0.87 (0.79, 0.95)
FG (per 10 mg/dL)	0.41 (0.39, 0.44)	1.80 (1.69, 1.91)	0.12 (0.10, 0.14)	1.12 (1.09, 1.15)	NS
HOMA-IR (per 10 unit)	NA	NA	NA	NA	1.87 (1.12, 3.11)
WBC count (per 1,000/μL)	NA	NA	NA	NA	1.10 (1.04, 1.16)
**MS component: Binary variable**					
Abdominal obesity^†^	0.92 (0.78, 1.07)	3.61 (2.98, 4.38)	0.42 (0.32, 0.53)	1.44 (1.23, 1.67)	1.38 (1.13, 1.69)
High BP^†^	0.43 (0.27, 0.59)	1.92 (1.54, 2.39)	0.35 (0.23, 0.46)	1.46 (1.22, 1.73)	NS
High TG^†^	0.44 (0.29, 0.59)	1.87 (1.55, 2.25)	0.48 (0.37, 0.59)	1.78 (1.53, 2.07)	NS
Low HDL-C^†^	0.27 (0.12, 0.42)	1.62 (1.34, 1.97)	0.23 (0.12, 0.34)	1.35 (1.16, 1.58)	1.29 (1.05, 1.59)
Impaired FG^†^	1.44 (1.30, 1.57)	7.80 (6.30, 9.64)	0.53 (0.42, 0.63)	1.67 (1.44, 1.95)	NS
IR^‡^	NA	NA	NA	NA	1.46 (1.13, 1.88)
Systemic inflammation^§^	NA	NA	NA	NA	1.34 (1.08, 1.67)

Data are odds ratio and coefficient (95% confidence interval) for binary and continuous outcomes, respectively. All analyses were adjusted for age, sex, smoking status, involuntary smoking status, physical activity, income, occupation status, education level, and resident region. ^†^Metabolic syndrome (MS) and MS components were defined according to the Joint Interim Statement clinical criteria with Korean-specific abdominal obesity criteria. ^‡^Insulin resistance (IR) was defined as the homeostasis model assessment of IR (HOMA-IR) > 3.06 (75^th^ percentile). ^§^Systemic inflammation was also defined as white blood cell (WBC) count >6,830/µL (75^th^ percentile). Only associations with *P* values < 0.05 are shown. WC, waist circumference; SBP, systolic blood pressure (BP); DBP, diastolic blood pressure; TG, triglyceride; HDL-C, high-density lipoprotein cholesterol; FG, fasting glucose. NS, not significant; NA, not applicable.

**Figure 2:**
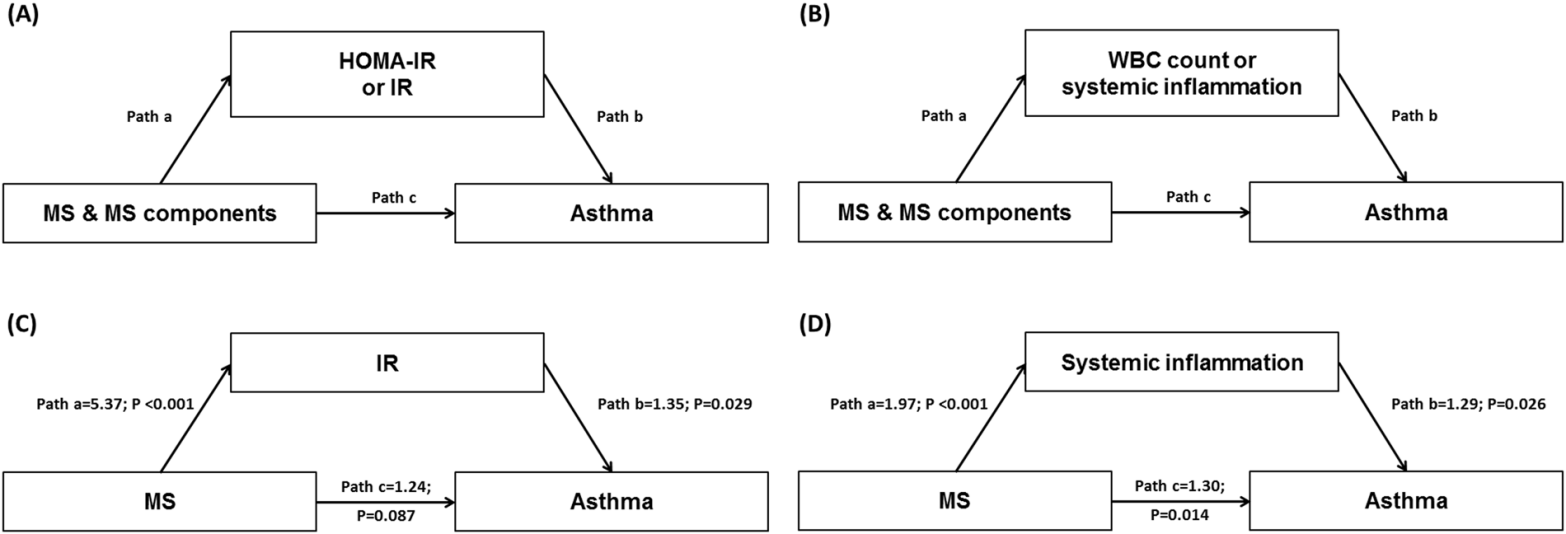
Mediation models of (**A**) HOMA-IR or IR and (**B**) WBC count or systemic inflammation in the relationship between MS and MS components, and asthma; examples† for mediation models of (**C**) IR and (**D**) systemic inflammation. IR was defined as HOMA-IR >3.06 (75^th^ percentile). Systemic inflammation was also defined as WBC count >6,830/µL (75^th^ percentile). Pathways and odds ratios are indicated with letters: pathway a represents the direct effect of the predictors (MS and MS components) on the mediators (HOMA-IR, IR, WBC count, and systemic inflammation); pathway b represents the direct effect of the mediators on the outcome (asthma); pathway c represents the direct effect of the predictors on the outcome. ^†^Indirect and mediation effects for these examples are shown in Table 3. MS, metabolic syndrome; HOMA-IR, homeostasis model assessment of insulin resistance (IR); WBC, white blood cell.

Mediation analyses revealed that greater waist circumference, lower HDL-C, abdominal obesity, low HDL-C, and MS were significantly related to asthma and at least one of the potential mediators (Fig. 2A,B; Table 3). MS was indirectly associated with asthma through IR but not directly associated with asthma, which resulted in the significant total effect of MS on asthma (Fig. 2C; Table 3). MS was directly and indirectly associated with asthma through systemic inflammation (Fig. 2D; Table 3). A substantial proportion of the association between MS and asthma was mediated by IR and systemic inflammation; the estimated mediation effect explained 39.8% and 69.8% of each association, respectively (Table 3).

**Table 3:** Mediation analyses for the effects of HOMA-IR and WBC count in the associations of WC, HDL-C and MS with asthma.

Mediator	Continuous variable				Binary variable			
Predictor	Total effect	Direct effect	Indirect effect	Percent mediated (%)	Total effect	Direct effect	Indirect effect	Percent mediated (%)
	HOMA-IR (per 10 unit)				IR^‡^			
Continuous variable								
WC (per 10 cm)	1.22 (1.08, 1.38)	1.19 (1.05, 1.35)	1.03 (0.99, 1.06)	9.0	1.50 (1.18, 1.91)	1.18 (1.04, 1.33)	1.28 (0.99, 1.64)	21.9
HDL-C (per 10 mg/dL)	0.85 (0.77, 0.95)	0.86 (0.77, 0.95)	0.99 (0.99, 1.00)	5.7	0.81 (0.72, 0.90)	0.86 (0.78, 0.96)	0.93 (0.88, 0.99)	20.8
Binary variable								
Abdominal obesity^†^	1.31 (1.04, 1.65)	1.25 (0.98, 1.58)	1.05 (1.00, 1.10)	11.0	1.82 (1.27, 2.62)	1.21 (0.95, 1.54)	1.51 (1.07, 2.13)	24.4
Low HDL-C^†^	1.28 (1.01, 1.62)	1.26 (0.99, 1.60)	1.02 (1.00, 1.03)	8.0	1.47 (1.12, 1.92)	1.24 (0.97, 1.57)	1.19 (1.03, 1.37)	26.1
MS^†^	1.35 (1.07, 1.71)	1.28 (1.01, 1.64)	1.06 (0.99, 1.12)	16.1	2.06 (1.32, 3.20)^¶^	1.24 (0.97, 1.59)^¶^	1.65 (1.05, 2.61)^¶^	39.8^¶^
**Predictor**	**WBC (per 1,000/mL)**				**Systemic inflammation** ^**§**^			
Continuous variable								
WC (per 10 cm)	1.26 (1.13, 1.40)	1.24 (1.11, 1.38)	1.02 (1.00, 1.04)	12.6	1.33 (1.18, 1.49)	1.25 (1.12, 1.39)	1.06 (1.00, 1.13)	60.1
HDL-C (per 10 mg/dL)	0.87 (0.79, 0.95)	0.87 (0.80, 0.96)	0.99 (0.99, 1.00)	3.9	0.84 (0.77, 0.93)	0.87 (0.80, 0.96)	0.97 (0.93, 1.00)	32.5
Binary variable								
Abdominal obesity^†^	1.38 (1.12, 1.68)	1.33 (1.08, 1.63)	1.04 (1.01, 1.06)	17.8	1.49 (1.20, 1.85)	1.35 (1.10, 1.65)	1.10 (1.01, 1.21)	68.1
Low HDL-C^†^	1.29 (1.05, 1.59)	1.26 (1.03, 1.56)	1.02 (1.00, 1.04)	6.6	1.38 (1.11, 1.71)	1.27 (1.03, 1.56)	1.09 (1.00, 1.18)	44.9
MS^†^	1.34 (1.09, 1.64)	1.28 (1.04, 1.57)	1.05 (1.01, 1.08)	17.8	1.54 (1.21, 1.95)^¶^	1.30 (1.05, 1.59)^¶^	1.19 (1.02, 1.39)^¶^	69.8^¶^

Data are odds ratio (95% confidence interval) with adjustments for age, sex, smoking status, involuntary smoking status, physical activity, income, occupation status, education level, and resident region. Only variables with P values < 0.05 of total effect are shown. ^†^Metabolic syndrome (MS) and MS components were defined according to the Joint Interim Statement clinical criteria with Korean-specific abdominal obesity criteria.^ ‡^Insulin resistance (IR) was defined as the homeostasis model assessment of IR (HOMA-IR) > 3.06 (75^th^ percentile). ^§^Systemic inflammation was also defined as white blood cell (WBC) count >6,830/µL (75^th^ percentile). ^¶^Mediation models are shown in Figure 2C and D. WC, waist circumference; HDL-C, high-density lipoprotein cholesterol.

## Discussion

This study showed that having MS is significantly associated with asthma defined as a self-reported wheezy episode in people aged ≥65 years. Among the MS components, abdominal obesity was most significantly related to asthma. However, the total number of MS components was also significantly associated with asthma. The presence of MS and the number of MS components were significantly associated with both IR and systemic inflammation. In the mediation analysis, it was observed that MS was significantly associated with asthma through IR and systemic inflammation.

As mentioned above, the association between MS and asthma in the elderly has been rarely investigated. Although several studies^12–14^ reported the link between MS and asthma, these studies did not include elderly patients. The HUNT study excluded those of age >65 years at follow-up^12^, the Nurses’ Health Study II included participants of age 26 to 46^13^ and the Norwegian study included people of age 14–60 years only^14^. It has not been clear if asthma in elderly patients and asthma in younger patients share a common pathophysiology and risk factors. Studies have shown that elderly asthma is phenotypically different from young asthma^7^. In fact, the overall average onset age of elderly asthmatics was around sixties in our study. Our study demonstrated the link between MS and asthma in the elderly, which could partially explain the mechanism of asthma in the elderly.

Our study showed that, among MS components, abdominal obesity is the most significantly related factor of asthma, which corresponds with previous studies. Several prospective cohort studies^14,15^ and cross-sectional studies^16^ have demonstrated that obesity is a predictor of asthma. It has not been clearly documented that components of MS other than obesity were significantly related to asthma. In one study, MS *per se* was not an independent predictor of asthma when body mass index was adjusted^15^. In our study, a low HDL-C was significantly associated with asthma, and a positive linear association between the number of MS components and the prevalence of asthma was observed. These findings suggest that MS-related factors other than abdominal obesity could contribute to the relationship between MS and asthma. In fact, MS components other than abdominal obesity such as hyperglycemia^17^, dyslipidemia^18^ and hypertension^19^ have been known to be risk factors of lower lung function or accelerated lung function decline.

The relationship between MS and asthma could be explained by several mechanisms: systemic and airway inflammation, IR, and oxidative stress. First, systemic inflammation leading to airway inflammation could have a role in the association between obesity and asthma. In obese patients with asthma, adipose tissues increased pro-inflammatory cytokines that lead to systemic inflammation with elevated levels of interleukin-6, tumor necrosis factor-α and leptin^20,21^. These alterations might contribute to macrophage proliferation and differentiation in lung tissues^20^ with subsequent airway inflammation. However, clinical studies did not support the association between obesity and increased inflammation in the airways of asthmatics^22^. Second, IR could contribute to the airway dysfunction. The association between IR and asthma in children and adults has been reported in studies^23,24^. IR is associated with skeletal muscle weakness, including the respiratory system^25^, by reducing glucose utilization and inducing abnormal fat metabolism in the muscle, which may impair mitochondrial adenosine triphosphate production. Thus, respiratory muscle dysfunction might result in the airflow obstruction seen in asthma^26^. Third, increased oxidative stress could have a role in the pathogenesis of asthma in obese participants. Obesity is associated with an increase in systemic oxidative stress^27^, and asthmatics are associated with a lower level of anti-oxidants^28^. However, it is unclear if the increase in airway oxidative stress is a consequence of changes in systemic oxidative stress caused by obesity^20^. We investigated how MS could be related to asthma. Our study evaluated IR by calculating the homeostasis model assessment of IR (HOMA-IR), and used the total number of white blood cell (WBC) as an indicator of systemic inflammation. The results revealed that the presence of MS and the number of MS components were positively associated with both IR and systemic inflammation, and IR and systemic inflammation were significantly associated with the prevalence of asthma (Table 2). The mediation analyses showed significant mediation effects through both IR and systemic inflammation in the relationship between MS and asthma (Table 3). In our study, systemic and airway oxidative stress was not evaluated.

Our study has several strengths. First, to our knowledge, we showed the significant association between MS and asthma in the elderly patients for the first time. Second, this study is based on data from a nation-wide survey that is representative of a large population. Third, mediation analyses provided a possible explanation of the link between MS and asthma, which supports the hypothesis that MS could be related to asthma through IR and systemic inflammation. We also admit limitations. Most of all, asthma was defined only based on self-reported wheezy episodes in our study. Although a questionnaire allowing the self-reporting wheezy episodes has been generally used in other studies for diagnosing asthma^6,29^, other various conditions can be related with wheezing sound. Of these, cases who could have chronic obstructive pulmonary disease were excluded based on spirometry results in our study, but we admit that our definition based on a self-report questionnaire could overlap with exercise-induced bronchoconstriction related to conditions other than chronic asthma such as obesity, lack of fitness or vocal cord dysfunction^30,31^. Second, the KNHANES survey did not measure airway inflammation or other systemic inflammatory biomarkers such as C-reactive protein. Oxidative stress was not measured. Third, our study is cross-sectional, and we could not verify the cause-effect relationship. There is a possibility that asthma increased the risk of MS in elderly patients. In fact, the mediation analysis showed a statistical significance in the analysis of the direction from asthma to MS (data not shown). Fourth, there are also many elderly asthmatics without MS, although we focused on the link between MS and elderly asthma. Interestingly, their onset ages were slightly different from those with MS. However, we could not clarify the characteristics and mechanisms of asthma in elderly without MS. Fifth, because characteristics of participants included in this study were significantly different from those excluded, our findings should be interpreted with caution.

The analysis using data from a nation-wide survey demonstrated a significant association between MS and asthma in elderly patients. MS might affect asthma through IR and systemic inflammation.

## Methods

For this study, cross-sectional data was acquired from KNHANES IV and V, which provide nationwide statistical data on the Korean population’s health and diet from January 2007 to December 2012^32^. KNHANES was administered by the Korean Ministry of Health and Welfare and the Korean Centers for Disease Control and Prevention. KNHANES implements a stratified multistage probability cluster sampling design. Sampling units were households based on geographic region, age, and gender groups. A health interview survey, nutrition interview, and physical examination were performed for each participant selected throughout South Korea. The study was exempt from ethical review in Seoul National University Hospital (IRB No. E-1601-017-730).

We selected participants who were 65 years or older. Those who did not undergo the laboratory tests for the measurement of IR or systemic inflammation, regarded as potential mediators in this study, were also excluded in the study. We additionally excluded the following participants: (a) chronic obstructive pulmonary disease (COPD) suspects (i.e., those with a ratio of forced expiratory volume in 1 second to forced vital capacity <70% and a previous or current diagnosis, treatment for COPD, or hospitalization due to COPD), (b) smokers who had a history of at least 10 pack-years, (c) participants with abnormal chest x-ray findings, and (d) participants who had a history of angina, myocardial infarction, stroke, or renal failure. The presence of asthma was determined by a positive response to the following two questions: “Have you had wheezing or whistling sound while breathing in the past 12 months?” and “Have you had wheezing or whistling sound while breathing during or after exercise in the past 12 months?” MS was defined according to the revised National Cholesterol Education Program criteria proposed by Joint Interim Society in 2009^33^ with the Korean-specific abdominal obesity definition^34^. Participants who had at least three MS components among the following were diagnosed as having MS: (i) waist circumference ≥90 cm for men or ≥85 cm for women^34^; (ii) systolic blood pressure ≥130 mmHg, diastolic blood pressure ≥85 mmHg, or treatment; (iii) triglycerides ≥150 mg/dL or treatment; (iv) HDL-C <40 mg/dL for men, or <50 mg/dL for women, or treatment, (v) fasting glucose ≥100 mg/dL or treatment. IR and systemic inflammation were regarded as potential mediators linking MS and asthma, because IR and systemic inflammation can be independent risk factors for asthma development *per se* in the context of MS condition^35^. Insulin sensitivity was estimated using HOMA-IR calculated as fasting insulin (µU/ml) × fasting glucose (mg/dl)/22.5^36^. Participants were diagnosed as IR if HOMA-IR was greater than 3.06 (75th percentile)^37^. WBC count greater than 6,830/µL (>75th percentile) was defined as systemic inflammation which was used as an indicator of systemic inflammation.

Among the above MS components, waist circumference was determined by measuring the participants’ smallest circumference between the iliac crest and the lower border of the rib. Blood pressure was taken three times in the seated position after 5 min rest and averaged. Blood samples for the analysis of triglyceride, HDL-C, and fasting glucose were collected after 8 hours fasting and analyzed using an ADVIA 1650 Chemistry Analyzer (Siemens, Deerfield, IL, USA). Insulin was measured using the immunoradiometric assay method with an auto-analyzer (Gamma counter, Hewlett Packard, USA, used for 2007 and January and February in 2008; 1470 WIZARD gamma-Counter, PerkinElmer, Finland, used for the other period in 2008 and 2009–2012). WBC count was measured using a Sysmex XE-2100D (Sysmex, Kobe, Japan) by laser flow cytometry.

**Figure 3:**
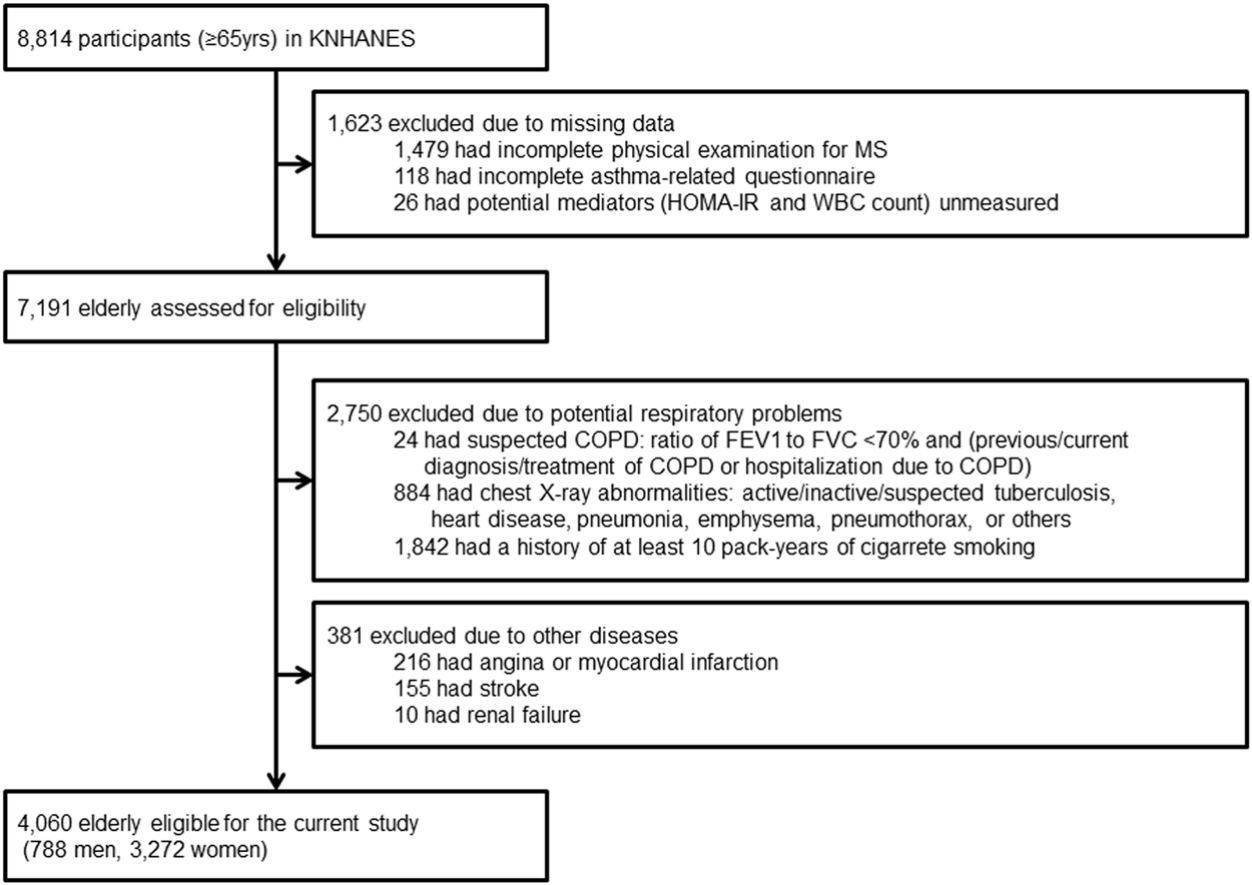
Flow of participants.

Among 8,814 elderly participants in the Korean National Health and Nutritional Examination Surveys (KNHANES) 2007–2012, 4,060 participants (788 men and 3,272 women) were eligible for our study (Fig. 3). Participants included in this study (n = 4,060) were significantly different from those excluded (n = 4,754) with respect to age (72.0 years and 73.4 years, respectively) and prevalence of MS (49.0% and 43.2%, respectively) and asthma (11.9% and 14.5%, respectively) (All *P* values < 0.001; data not shown). Significant sex-MS interactions were not found for asthma, which indicates that sex does not modify the relationship between MS and asthma. Men and women were, therefore, not analyzed separately.

Participants were divided according to the MS status and analyzed for participant characteristics. Continuous and categorical variables were compared between two groups using the Student t test and Chi-square test, respectively. Multivariable logistic regression analyses were used to ascertain the associations of MS, MS components, IR, and systemic inflammation with asthma. Multivariable generalized linear regression analyses were used to ascertain the associations of MS and MS components with HOMA-IR and WBC count. Covariates included in multivariable regression models were age (65–69, 70–74, ≥75 years), sex, smoking status (ever-smoker, non-smoker), involuntary smoking status (involuntary smoker, non-involuntary smoker), physical activity, income (≥, <$2,000/month), occupation status (working, retired), education level (≥, <middle school), and resident region (urban, rural).

Mediation analyses using the Karlson-Holm-Breen decomposition method (Stata 14.0; Stata Corp., College Station, TX, USA) were conducted to determine if the associations of MS and MS components with asthma were explained by HOMA-IR, IR, WBC count, or systemic inflammation. Mediation analyses were utilized only when (1) the predictor (MS and MS components) was significantly associated with the mediator (HOMA-IR, IR, WBC count, and systemic inflammation); (2) the mediator was significantly associated with the outcome (asthma); (3) the predictor was significantly associated with the outcome in the absence of the mediator; and (4) the association between the predictor and outcome was attenuated when the mediator was included in the model^38^. Multivariable logistic and linear regression analyses were used to determine these associations. Percent mediated was measured using the ratio of the absolute value of the indirect effect [|(coefficient of pathway a) × (coefficient of pathway b)|] to the absolute value of the total effect of metabolic components on the outcome [|(coefficient of pathway a) × (coefficient of pathway b)| + |(coefficient of pathway b)|] in Fig. 2. We did not control for Type-I errors due to multiple comparisons, because this study was exploratory for potential mediators in nature. SAS 9.4 (SAS Institute Inc., Cary, NC, USA) software packages were used to perform all statistical analyses other than the mediation analyses. *P* values < 0.05 were considered statistically significant.
